# Delivery of Surgical Care by Local Actors in Chronic Conflict Settings: The Example of Bor State Hospital, South Sudan

**DOI:** 10.1002/wjs.70292

**Published:** 2026-02-27

**Authors:** Majok Philips Matiop, Amila Sanjiva Ratnayake, Anuradhi Lanka Wijekoon, Hannah B. Wild

**Affiliations:** ^1^ Medical Director Bor State Hospital Bor Jonglei South Sudan; ^2^ Army Hospital Colombo Colombo Sri Lanka; ^3^ Uniformed Services University of the Health Sciences Bethesda Maryland USA; ^4^ Department of Surgery University of Washington Seattle Washington USA; ^5^ Explosive Weapons Trauma Care Collective (EXTRACCT) Seattle Washington USA

**Keywords:** chronic conflict, complex humanitarian emergencies, emergency critical and operative care, essential surgical care, humanitarian assistance, South Sudan

## 1 Background

The surgical realities of armed conflict are starkly concrete. Regardless of the specific geopolitical objectives playing out at a tactical level, on a human scale these events reach the first aid station or operating theater where taxed medical personnel work with minimal resources to care for the wounded. This editorial perspective examines the profound and often overlooked surgical implications of chronic armed conflict using a case example of Bor State Hospital in South Sudan. South Sudan has been ravaged by decades of conflict. Even in the absence of active fighting, the health system struggles to meet the surgical needs of the population given strained resources, limited trained personnel, and damaged health infrastructure.

Bor Town, a city of approximately 25,000 people and the capital of Jonglei State, is emblematic of the brutal cycles of violence that have characterized South Sudan's history. Bor was the site of one of the first major battles in the civil war that erupted in December 2013 which was fought over for a month, during which time the city changed hands four times. As of late 2025, South Sudan is experiencing an alarming resurgence in fighting, with the highest number of war‐wounded at ICRC‐supported hospitals in the country since 2018 [1].

### 1.1 Impact of Chronic Conflict on the Delivery of Surgical Care at BSH

Armed conflict results in the destruction of healthcare infrastructure in already‐fragile exceptions and the case of Bor State Hospital (BSH) was no exception. BSH was ransacked and looted, losing basic equipment such as ultrasounds, laboratory materials, and X‐ray machines, in addition to the exodus of trained healthcare personnel. National instability has turned South Sudan into a state grappling with basic primary health care, let alone the infrastructure required to provide safe surgical care. In addition to chronic shortages of essential medicines and supplies at BSH, the health workforce is critically short. In Bor, healthcare staff are often unpaid for months and work under constant threat of violence, which drives migration and makes retention extremely difficult. As of October 2025, there were only four general practitioners and one general surgeon serving in BSH. Bor has been the site of repeated mass casualty events, which leave the hospital overwhelmed as it lacks the capacity and resources to handle the influx of patients [2].

The current state of surgical care at BSH reflects a system operating under extreme chronic strain, primarily driven by the overlapping effects of internal and regional conflict. BSH operates a 200‐bed medical facility that offers a range of services including general medicine, pediatrics, maternal and child health, and surgical care. The surgical department has 55 beds and currently performs an average of 420 procedures annually, ranging from minor interventions (e.g., incision and drainage, hernia) to major surgeries (e.g., craniotomy, trauma laparotomy). The surgical team consists of dedicated all‐local staff including one (general) surgeon, one general practitioner, three nurse anesthetists, and seven nurses. There is one major and one minor operating room. The minor operating theater lacks basic equipment such as an anesthesia machine, suction drain, and hemodynamic monitoring therefore is used for procedures performed under local or regional anesthesia (e.g., laceration repair, complex dressings changes, debridements). Local and regional anesthesia as well as ketamine are often the only available options due to the lack of reliable supply chains for maintenance of anesthesia including vaporizors, oxygen and nitrous regulators, and general anesthetic agents. With respect to the war‐wounded, many patients arrive with gunshot wounds and blast injuries often after delays of several days due to insecurity, distance, and poor roads, especially during the rainy seasons. The small surgical team is barely incentivized with gaps in salaries and often exhausted, requiring that care often be focused on life‐saving surgery only.

Delivering surgical care in chronic conflict settings, as seen across South Sudan and similar low‐resource regions affected by protracted war, involves overcoming profound systemic challenges. South Sudan faces a critical shortage of qualified healthcare professionals, exacerbated by uneven distribution outside the capital city of Juba and high attrition rates, particularly in rural regions. Only 44% of South Sudan's population lives within 5 km of any health facility, and just 68% of facilities are functional nationwide [3]. Despite this BSH is the only referral hospital in Jonglei and also serves the Greater Pibor Administrative Area (GPAA), Yirol in Lakes State, and Panyijiar in Unity State.

### 1.2 The Presence of Humanitarian Actors in Chronic Conflict Settings

There is a substantial presence of international non‐governmental organizations (NGOs) in South Sudan such as ICRC and Medicine Sans Frontières (MSF) assisting to recover and maintain surgical capacity [4]. The World Health Organization (WHO) is a technical partner to the Ministry of Health (MOH) and provides operational and technical support to BSH. United Nations Children's Fund (UNICEF) complements these efforts by implementing a World Bank project focused on providing sustained health system support including provision of regular staff incentives and quarterly essential medical supplies [5]. The United Nations Mission in South Sudan (UNMISS) Level 2 hospital operated by the Sri Lankan contingent provides indirect support for life‐ and limb‐saving emergency interventions, facilitation of referrals, and periodic donation of medical and surgical supplies.

While these contributions are indispensable in preventing system collapse, they remain largely centered around humanitarian rather than local actors, time‐limited, and siloed. The chronic conflict in Bor underscores the need for a transition toward coordinated sustainable capacity building, locally‐anchored workforce development, and resilient national health system strengthening, ensuring that humanitarian support translates into durable improvements in emergency critical and operative (ECO) care outcomes [6].

### 1.3 Recommendations

The experience of the authors at Bor State Hospital is believed to represent conditions and challenges faced by many local healthcare providers in chronic conflict settings. We believe some of the following concrete steps could be taken to address these gaps:

1. Investment in local training: Jonglei Health Science Institute (JHSI) trains mid‐level health cadres (clinical officers and general practitioners with basic surgical skills) within Bor. Enhancing these training to involve more specialties such as anesthesia and radiology technicians will result in long sustainable benefits for ECO care. Attention should also be given to task‐shifting training programs and dedicated postgraduate programs that can expand the workforce in more remote regions outside of Bor [7].
2. Strategic selection of essential equipment: In chronic conflict settings, prioritizing durable equipment that is adapted to the local conditions and can be maintained by local actors is crucial for ensuring the resilience and sustainability of health systems and infrastructure.
3. Implement context‐specific clinical guidelines and trauma care capacity assessment tools: Context‐appropriate guidelines for clinical care and implementation of quality improvement interventions should align with the unique dynamics of each chronic conflict setting. This includes direct partnership with local actors in order to understand local stakeholders and conflict dynamics. Numerous efforts exist to provide standardized yet context‐appropriate clinical care and capacity assessments in low‐resource conflict settings, including the Explosive Weapons Trauma Care Collective (EXTRACCT) Clinical Practice Guidelines (CPGs) for blast injury in low‐resource settings and the INTACT‐B tool to assess existing trauma care capacity [8, 9].
4. Standardized data collection: Investing in context‐appropriate data registries with minimal data sets using variables that are available even in highly resource‐constrained settings will guide appropriate performance improvement efforts, help improve patient outcomes, and facilitate monitoring of the impact of humanitarian and MOH projects [10].

## 2 Conclusion

The experience of Bor State Hospital in Jonglei State, South Sudan, exemplifies challenges faced by local healthcare systems in chronic conflict settings that limit access to safe surgical care for both war‐related injuries as well as non‐trauma emergencies among the local population. While efforts exist at numerous levels to address these gaps, at present, disproportionate emphasis on humanitarian response by international NGOs with limited strategic planning for durably strengthening local capabilities can detract from longterm solutions. Areas of priority included closer partnership with local stakeholders including surgical education and trauma training programs as well as contextually‐adapted care standards (e.g., minimum data registries, clinical practice guidelines, trauma care capacity assessment tools). Prioritizing capacity‐building within the local health system requires years of commitment but may meaningfully improve surgical care in chronic conflict settings.

## Author Contributions

**Majok Philips Matiop:** supervision, writing – review and editing, project administration. **Amila Sanjiva Ratnayake:** writing – review and editing, writing – original draft, conceptualization, investigation, project administration. **Anuradhi Lanka Wijekoon:** writing – original draft, writing – review and editing, project administration. **Hannah B. Wild:** supervision, writing – review and editing, project administration.

## Conflicts of Interest

The authors declare no conflicts of interest.

## Data Availability

Data sharing not applicable to this article as no datasets were generated or analysed during the current study.
